# Development of an *in Vitro* Potency Assay for Anti-anthrax Lethal Toxin Neutralizing Antibodies

**DOI:** 10.3390/toxins4010028

**Published:** 2012-01-19

**Authors:** Gail Whiting, Michael Baker, Sjoerd Rijpkema

**Affiliations:** Division of Bacteriology, National Institute of Biological Standards and Control, HPA, Blanche Lane, Potters Bar, Hertfordshire EN6 3QG, UK; Email: michael.baker@nibsc.hpa.org.uk (M.B.); sjoerd.rijpkema@nibsc.hpa.org.uk (S.R.)

**Keywords:** anthrax, lethal toxin, toxin neutralisation

## Abstract

Lethal toxin (LT) of *Bacillus anthracis* reduces the production of a number of inflammatory mediators, including transcription factors, chemokines and cytokines in various human cell lines, leading to down-regulation of the host inflammatory response. Previously we showed that the reduction of interleukin-8 (IL-8) is a sensitive marker of LT-mediated intoxication in human neutrophil-like NB-4 cells and that IL-8 levels are restored to normality when therapeutic monoclonal antibodies (mAb) with toxin-neutralising (TN) activity are added. We used this information to develop cell-based assays that examine the effects of TN therapeutic mAbs designed to treat LT intoxication and here we extend these findings. We present an *in vitro* assay based on human endothelial cell line HUVEC jr2, which measures the TN activity of therapeutic anti-LT mAbs using IL-8 as a marker for intoxication. HUVEC jr2 cells have the advantage over NB-4 cells that they are adherent, do not require a differentiation step and can be used in a microtitre plate format and therefore can facilitate high throughput analysis. This human cell-based assay provides a valid alternative to the mouse macrophage assay as it is a more biologically relevant model of the effects of toxin-neutralising antibodies in human infection.

## 1. Introduction

*Bacillus anthracis* is a spore-forming bacterium, which occurs naturally in soils throughout the world and causes the disease anthrax. *B. anthracis* produces two binary toxins; edema toxin (ET) and lethal toxin (LT). ET is composed of protective antigen (PA) and edema factor (EF) whereas LT comprises PA and lethal factor (LF) [1,2]. PA binds to cell surface receptors and following cleavage by furin, polymerises into a heptameric structure that can bind EF and LF and promote their entry into the cell. EF is a calmodulin-dependent adenylate cyclase that increases intracellular cAMP, culminating in edema [3]. LF is a zinc metalloprotease that cleaves the amino terminus of the mitogen-activated protein kinase (MAPK) kinases, preventing binding to downstream mitogen activated protein kinases such as extracellular regulated kinase (ERK) or p38, leading to the complete inhibition of the MAP kinase signalling pathway and, ultimately, cell cycle arrest and cell death [4,5,6].

Inhalational anthrax is a potent bioterrorism threat because the anthrax spores are stable, relatively easy to aerosolize and disperse and have the potential to infect a large number of people. In addition the early symptoms of anthrax disease are frequently non-specific and diagnosis of anthrax is difficult until the disease progresses to the later stages. The result is that the fatality rate for inhalational anthrax is estimated to be between 45% and 90%, even after the use of aggressive antibiotic treatment.

Post-exposure vaccination is unlikely to be protective because of the delay between exposure to anthrax and development of immunity. Recently, several therapeutic antibody preparations have been developed with the aim to treat inhalational anthrax disease. These include human or humanised monoclonal antibodies (mAbs) and human polyclonal antibodies which react primarily with PA, but also EF and LF [7,8]. Blocking the effects of the toxins is central for host protection against anthrax and there is significant evidence that protection is effected by anti-toxin antibody responses [9,10].

For the evaluation of therapeutic antibody preparations it is essential to determine the capacity of the antibody preparations to neutralise anthrax toxins. *In vitro* toxin neutralisation (TN) assays based on murine macrophage cell lines J774A.1 and RAW264.7 are frequently used and cell survival is determined following exposure to LT or to a mixture of LT and an antibody of choice [11,12,13]. A CHO cell-based assay has also been used to assess anti-PA therapeutic monoclonal antibody levels by measuring reduction in ET-induced cAMP levels [14]. 

The murine macrophage cell lines used at present in LT assays are killed by the toxin whereas most human cells are resistant and hence can be used to model the *in vivo* effects of the toxin during human infection. Previously, we used the human neutrophil-like cell line NB-4 to study effects of LT exposure [15,16]. Cell death was not observed, however intoxicated NB-4 cells produced less mRNA of pro-inflammatory cytokines and transcription factors as well as lower levels of constitutively expressed proteins that are essential for cellular homoeostasis such as actin-related protein, ATP synthase β chain and high-mobility group box chromosomal protein 1 (HMGB1) [15,16]. These genes, with the exception of HMGB1, have been identified previously as markers for LT mediated toxicity in various human immune cells [15,17,18,19,20,21,22]. Reductions in mRNA and protein levels of pro-inflammatory cytokines such as IL-8 in NB-4 cells provided us with relevant, highly significant and sensitive biological markers for LT intoxication [15]. Indeed neutralisation of LT by an anti-LF monoclonal antibody restored IL-8 levels in cell culture supernatants [15]. However, this cell type is not easily adapted to a microtitre plate format, often used for routine testing: cells are non-adherent and require a time-consuming differentiation step. The aim of this study was to develop a high throughput assay to detect TN activity of anti-PA and anti-LF antibody preparations. For this purpose we replaced the NB-4 cells with endothelial cells. This cell type is adherent and does not require differentiation, making it suitable for adaptation to a microtitre plate format. 

Here we report on a TN assay that measures the activity of LT on the human endothelial cell line HUVEC jr2. We show that IL-8 and IL-6 production are depressed following LT exposure and restored to normality when toxin neutralising mAbs are added to the cells.

## 2. Materials and Methods

### 2.1. Anthrax Lethal Toxin

Recombinant anthrax toxin PA and LF were obtained from List Biological Laboratories (Campbell, CA, USA). Antigens were snap frozen in liquid nitrogen and stored at −70 °C in 10 µL aliquots at a concentration of 1 mg/mL.

### 2.2. Antibody Preparations

The relevant characteristics of antibody preparations used in this study are given in Table 1.

**Table 1:** Toxin-neutralising (TN) monoclonal antibodies used in this study.

ID (Specificity)	Source	Species	TN activity [Reference]
IQNLF (anti-LF)	IQ Therapeutics, The Netherlands	Human	50% at 15 ng/mL [9]
IQNPA (anti-PA)			50% at 50 ng/mL [9]
IQNLF and IQNPA			>50% at 35 ng/mL [9]
9A11 (anti-LF)	Duke University, North Carolina, USA	Mouse	50% at 200 ng/mL [10]

### 2.3. Cell Culture

Endothelial cells were derived from a pooled stock of HUVEC jr2 cells [23]. Cells from a single working cell bank were used between passage 2 and passage 13 and were seeded at 0.8 × 10^6^ cells per 75 cm^2^ flask and maintained in EGM-2 medium (Lonza, Slough, UK). Neutrophil-like NB-4 cells were derived from the promyelotic leukaemia cell line NB-4 and differentiated by exposure to 9-*cis*-retinoic acid, Vitamin D_3_ and granulocyte colony stimulating factor [24]. NB-4 cells were maintained in RPMI-1640 supplemented with 10% foetal bovine serum (FBS) and 1% L-glutamine (all from Sigma-Aldrich, Dorset, UK). Cells were maintained in a 95% humidified atmosphere containing 5% CO_2_.

### 2.4. Analysis of Gene Expression in HUVEC jr2 Cells

HUVEC jr2 cells were exposed to 40 nM LT (40 nM PA and 11 nM LF) for 2 and 4 h in line with previous gene expression analysis in NB-4 cells [15,16]. Untreated cells or cells treated with LF or PA were used as controls. Total RNA was extracted from cells (Qiagen RNeasy kit, Crawley, UK) after treatment and any contaminating DNA was removed by Dnase treatment (Applied Biosystems, Warrington, UK). cDNA was generated using Superscript II Rtase (Invitrogen, Paisley, UK) using random primers (Promega), following the manufacturer’s instructions. All gene expression primers and the universal PCR mastermix were from Applied Biosystems and used according to the manufacturer’s guidelines. β-actin was used as an endogenous control. Fifty nanograms of cDNA were added per reaction and RT PCRs were performed on an Applied Biosystems 7500 Fast instrument under standard cycling conditions. Quantification was performed using the comparative Ct method where Ct value was defined as threshold cycle of PCR at which amplified product was detected and normalised to the Ct value of housekeeping gene β-actin. Data are shown as fold change in expression relative to the expression level of specific mRNA in untreated cells at a given time point, which have an arbitrary value of 1. The results represent data combined from two separate cell experiments, with PCRs performed twice and using duplicate or triplicate wells.

### 2.5. TN Assay Based on HUVEC jr2 Cells

After passaging, rinsing with HEPES-buffered saline solution (Lonza) and trypsinization with Trypsin-EDTA (Lonza), HUVEC jr2 cells were resuspended in EGM-2 medium to 0.5 × 10^5^ cells per ml and 10^5^ cells were added per well into a 24 well plate and were incubated for 24 h at 37 °C in 5% CO_2_ for cells to attach and form a monolayer. After 24 h the spent culture medium was removed from the wells and replaced with fresh EGM-2 only (untreated control well) or EGM-2 containing LT and in some cases 1 µg/mL lipopolysaccharide (LPS) from *Escherichia coli* (Sigma). LT concentrations ranged from 10 nM PA, 1 nM LF to 0.2 nM PA, 0.02 nM LF with the highest concentration used in development of NB-4 based TN assay [15]. LT-specific antibody diluted to 100 µg per ml in sterile PBS was added at concentrations between 50 and 1000 ng/mL to appropriate wells. The cells were then incubated for a further 24 h and culture supernatant was removed to be assayed for IL-8 by ELISA. Human IgG1a (Sigma) was used as a negative control for human mAb preparations. Human and mouse mAbs (500 ng/mL) were added to untreated cells to verify absence of non-specific stimulation of IL-8 production.

### 2.6. Cell Viability Assay

A non-radioactive cell proliferation assay which measures colour change due to conversion of formazan to MTT (3-(4,5-Dimethylthiazol-2-yl)-2,5-dipheyltetrazolium bromide) was used as a marker for the number of live cells per well according to the manufacturer’s protocol (Promega, Southampton, UK). Absorbance readings were compared to untreated control to provide an estimate of cytotoxicity.

### 2.7. IL-6 and IL-8 ELISA

Culture medium was assayed for the presence of IL-6 and IL-8 protein using human IL-6 and IL-8 capture ELISA kits (R and D Systems, Abingdon, UK) according to the manufacturer’s instructions. Cell culture supernatants were diluted 2–16-fold with reagent diluent. The data represent the mean of two cell experiments, assayed twice and duplicate samples for each experiment.

### 2.8. Processing and Analysis of ELISA Data

The amount of IL-6 and IL-8 in the samples was calculated relative to the IL-6 and IL-8 standard by parallel line assay. The calculations were performed using the current version of Combistats statistical analysis program [25]. OD readings were corrected for background and were required to be within a range of 2.25–0.20. Only results within the linear dose range of the curve were used for the potency calculation. Potency values derived from the parallel line assay were converted to IL-8 content in pg/mL. Statistical analysis of ELISA data was carried out by the analysis of variance with Dunnett’s test.

## 3. Results

### 3.1. Lethal Toxin Rapidly Alters Gene Expression in Endothelial Cells

**Figure 1:**
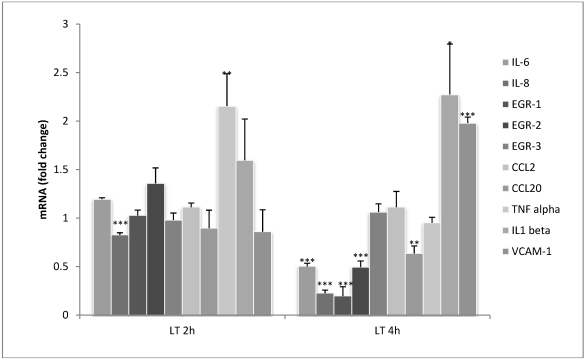
Lethal toxin (LT) rapidly alters the expression of chemokines, cytokines and transcription factors in HUVEC jr2 cells after 2 and 4 h. Cells were exposed to 40 nM LT (40 nM PA + 11 nM LF) or left untreated. The data presented here compares the LT-treated cells to untreated cells at 2 and 4 h and the fold change for both 2 and 4 h untreated cells is 1. Data from two independent experiments are shown as relative expression ± SEM. *** *p* < 0.001; ** *p* < 0.01; * *p* < 0.05.

Gene expression of six cytokines, three transcription factors (early growth response genes (*egr*) 1-3) and vascular cell adhesion molecule (VCAM)-1 were examined in HUVEC jr2 cells after exposure to LT. RT-PCR results show that the effect of LT exposure was more pronounced at 4 h than at 2 h (Figure 1). At 4 h, significant decreases in mRNA were seen for IL-6, IL-8 and CCL20; this pattern concurred with previous findings in NB-4 cells by Barson *et al*. [15]. 

Some differences in the cellular response after exposure to LT were observed between the two cell types however. IL-6 mRNA could not be detected in NB-4 cells and CCL2 (monocyte chemotactic protein-1) gene expression was down-regulated in NB-4 cells but in endothelial cells no change was observed. *Egr-1* and *egr-2* were down-regulated in HUVEC jr2 cells whereas *egr-3* remained unchanged. Down-regulation of *egr1* in endothelial cells after LT exposure was also reported by Rolando *et al*. [22]. In NB-4 cells, *egr* 1–3 were down-regulated [15]. Levels of IL-1β mRNA increased after 4 h and TNF-α mRNA showed a transitory increase in expression at 2 h which had fallen back to normal levels by 4 h. Both genes are key mediators of the acute phase response and have previously been shown to be down-regulated in murine macrophages and NB-4 cells exposed to LT [15,26].

The effect of LT on the mRNA expression of *vcam-1* was examined and our results show a 2-fold increase which is in line with findings by Warfel and D’Agnillo [27]. VCAM-1 expression is thought to be associated with vascular leakage which is caused by disruption of the vascular endothelium, a key feature of anthrax pathogenesis in clinical cases and in animals injected with LT [27,28,29]. 

### 3.2. LT Reduces Cytokine Production in Endothelial Cells

Spent culture medium from cells treated with LT was analysed for the presence of IL-6 and IL-8 proteins. In one series of experiments the HUVEC jr2 cells were stimulated with lipopolysaccharide (LPS) to ensure that any changes in cytokine production following LT treatment were of sufficient magnitude. LPS induces cytokine production via activation of Toll-like receptors in a similar manner to *B. anthracis* [30]. 

**Figure 2:**
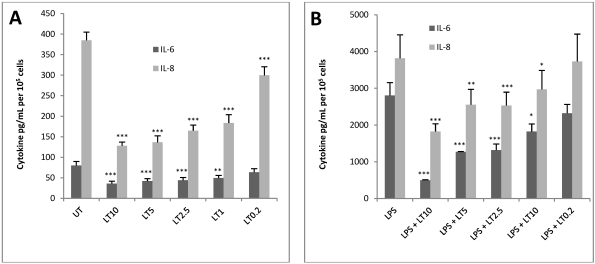
LT decreases protein levels of IL-6 and IL-8 in unstimulated (**A**) and LPS-stimulated (**B**) HUVEC jr2 cells. Cells were treated with concentrations of LT (LT10–10 nM PA and 1.0 nM LF, LT5–5 nM PA and 0.5 nM LF, LT2.5–2.5 nM PA and 0.25 nM LF, LT1–1 nM PA and 0.1 nM LF, LT0.2–0.2 nM PA and 0.02 nM LF) and/or LPS (1 μg/mL) for 24 h and conditioned medium was collected and analysed by ELISA. Data shown as mean ± SEM (*n* = 4). *** *p* < 0.001; ** *p* < 0.01; * *p* < 0.05 compared to untreated control (unstimulated or LPS-stimulated as appropriate).

Results showed that for HUVEC jr2 cells treatment with LT ranging from 10 nM PA and 1 nM LF to 0.2 nM PA and 0.02 nM LF reduced the level of IL-6 and IL-8 irrespective of LPS treatment. In unstimulated cells the production of IL-6 was reduced significantly except when treated with the lowest concentration (Figure 2A). In LPS-stimulated cells, LT caused reductions of IL-6 of between 1.2 to 5.5-fold however this reduction was not significant at the lowest LT concentration (Figure 2B).

Levels of IL-8 protein were similarly reduced by LT treatment with LPS-stimulated cells with decreases of 1.3 to 2-fold for all but the lowest concentration. In unstimulated cells the reduction was between 1.25 to 3-fold. The lowest concentration of 0.2 nM PA and 0.02 nM LF reduced IL-8 by a significant degree for the unstimulated cells only (Figure 2A). Both the unstimulated HUVEC jr2 cells and those stimulated with LPS gave a clearly defined dose response with the concentrations of LT used for both IL-6 and IL-8 and statistical analysis revealed that the decline in IL-6 and IL-8 was significant. The decrease in IL-8 production by LT-treated cells was not likely to be due to a decrease in cell viability as it ranged from 93% for LT10 to 97% for LT0.2, compared to the untreated control.

### 3.3. Application of HUVEC jr2 Cells in Toxin Neutralisation Assay

We have demonstrated previously that the production of IL-8 by NB-4 cells is a very sensitive marker of LT-mediated intoxication. Levels of IL-8 are depressed in the presence of LT but are restored to normality when toxin neutralising mAbs are added to the cells [15]. This formed the basis of a TN assay to confirm the potency of both anti-PA and anti-LF mAbs. However, the use of NB-4 cells in this assay has two disadvantages. NB-4 cells are not adherent and thus are not suitable for culture in a microtitre plate environment and NB-4 cells require differentiation to acquire a neutrophil-like phenotype. Thus a TN assay based on endothelial cells should be easier to perform and allow for multiple samples to be tested concurrently.

A modified assay procedure was developed for the endothelial cell line HUVEC jr2. After exposure to LT, this cell line showed a more marked decrease in the production of IL-8 compared to IL-6 (Figure 2). Therefore we chose to use IL-8 as a marker of LT intoxication in HUVEC jr2 cells as the basis of the TN assay.

**Figure 3:**
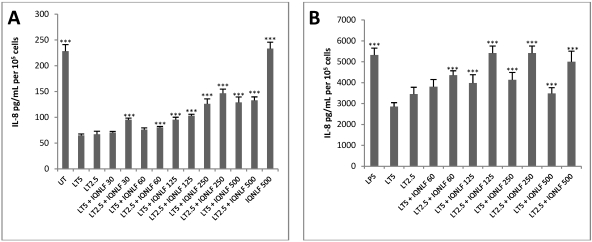
Restoration of IL-8 protein production by anti-LF mAb IQNLF in (**A**) unstimulated HUVEC jr2 cells and (**B**) LPS stimulated HUVEC jr2 cells exposed to LT. UT or LPS-control, LT5–5 nM PA and 0.5 nM LF, LT2.5–2.5 nM PA and 0.25 nM LF. Antibody concentrations were from 30 to 500 ng/mL for IQNLF. Data shown as mean ± SEM (*n* = 4). *** *p* < 0.001 compared to LT treated cells.

For the assay, 10^5^ cells were seeded into each well of a 24-well plate. LT and the anti-LF mAb IQNLF were added and plates were incubated at 37 °C for 24 h. The spent culture medium was collected and IL-8 production was measured by ELISA. Levels of IL-8 protein were reduced when LT was added and addition of IQNLF resulted in a restoration of IL-8 production to normal levels. This effect was seen in both LPS-stimulated and unstimulated cells (Figure 3) and in both cases the decline and restoration of IL-8 following treatments was statistically significant. In unstimulated cells treated with LT, restoration of IL-8 showed a good dose response over a range of mAb concentration of 30–500 ng/mL thus removing the requirement for prior stimulation with LPS in the TN assay (see also 3.2). To rule out the possibility that addition of the mAb into the TN assay could non-specifically induce changes in IL-8 we added IQNLF at 500 ng/mL and our results showed that there was no significant difference in IL-8 production compared to the control.

### 3.4. TN Antibodies Restore IL-8 Production in LT-Treated HUVEC jr2 Cells

**Figure 4:**
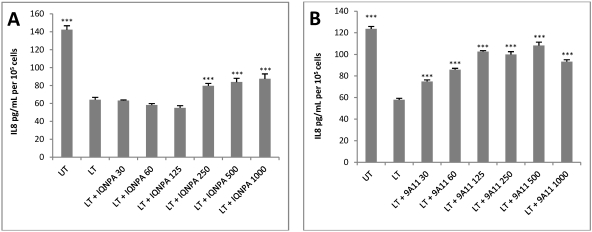
Restoration of IL-8 protein production by anti-anthrax mAbs (**A**) IQNPA and (**B**) 9A11 in HUVEC jr2 cells exposed to LT. UT–untreated control, LT–2.5 nM PA and 0.25 nM LF. Antibody concentrations ranged from 30 to 1000 ng/mL for IQNPA and 9A11. Data shown as mean and SEM (*n* = 4). *** *p* < 0.001; ** *p* < 0.01 compared to LT treated cells.

Once we had established the suitability of HUVEC jr2 cells for use in a microtitre plate format, we examined the ability of other TN mAbs to restore levels of IL-8 production in unstimulated LT-treated cells. We observed that the anti-PA mAb IQNPA was able to restore IL-8 production, at concentrations of 250 to 1000 ng of mAb per mL, although IL-8 levels were not restored to 100% compared to the untreated control (Figure 4A). Anti-LF mAb 9A11 completely restored IL-8 production in HUVEC jr2 cells at concentrations of 250, 500 and 1000 ng/mL (Figure 4B). HUVEC jr2 cells did not produce higher levels of IL-8 following exposure to 500 ng/mL of mAbs IQNPA or 9A11 (results not shown). 

**Figure 5:**
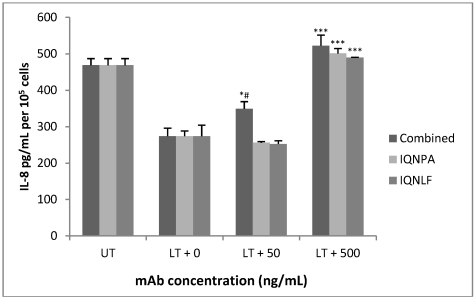
Efficacy of IQNLF and IQNPA mAbs in restoring IL-8 protein production by HUVEC jr2 cells exposed to LT (5 nM PA and 0.5 nM LF). * *p* < 0.05 for LT + 50 (25 ng IQNLF and 25 ng IQNPA per mL) compared to LT + 0 (no antibody treatment), # *p* < 0.05 (25 ng IQNLF and 25 ng of IQNPA combined compared to 50 ng of IQNLF or IQNPA), *** *p* < 0.001 for LT + 500 (500 ng IQNLF or IQNPA per mL or 250 ng of IQNLF and 250 ng of IQNPA per ml) compared to LT + 0.

When anti-PA mAb IQNPA and anti-LF mAb IQNLF were combined for use in the TN assay (25 ng/mL of each) we found that their presence increased IL-8 levels significantly but that a single mAb did not restore IL-8 production at this concentration of 50 ng of mAb per mL. IL-8 levels were restored completely in the presence of 500 ng per ml of each antibody and also with the IQNLF and IQNPA combination (Figure 5).

## 4. Discussion

Here we demonstrate that human endothelial cells can be used in a microtitre plate format to detect LT mediated toxicity and TN activity of mAbs. Endothelial cells have two major advantages over NB-4 cells which allow the development of this application: cells are adherent, making these cells more suitable for culture in a microtitre plate format and cell differentiation and stimulation by LPS can be omitted making routine culture of this cell line simpler and less costly. Like NB-4 cells, HUVEC jr2 cells proved very sensitive to the effects of LT [15], which manifested itself by a decrease in mRNA transcription of various cytokines (IL-6 and IL-8) and transcription factors, such as *egr1*, a master regulator of transcription. The down-regulation of *egr1* by LT has been reported for other endothelial cell lines exposed to LT or wild-type *B. anthracis* [22,31]. Lower levels of *egr1* lead to the formation of actin cables, which were associated with barrier dysfunction [22,32]. Our results indicate that the *egr* family of transcription factors is regulated differently in endothelial cells compared to neutrophil-like NB-4 cells. Thus it is likely that LT has cell-type specific effects which in turn affect different sets of genes regulated by MAP kinases [33]. The gene expression of several regulators of the actin cytoskeleton such as cortactin and rhophilin-2 are modulated by LT in endothelial cells [22] and LT also directly blocks the signalling pathways controlling actin regulators [18,19]. 

Abnormal or dysregulated VCAM-1 activation is linked to vasculitis and inflammatory disorders, both of which are key features of anthrax pathogenesis. We examined the effect of LT on *vcam1* and our results show a 2-fold increase in expression in line with findings by Steele *et al*. [34] and Warfel and D’Agnillo [27]. 

In addition, several reports in the literature have shown conclusively that endothelial cells are a target for LT and their deterioration plays an important role in the progression of infection [2,17,28,31]. 

IL-8 is an important mediator of inflammation which acts both as a cytokine and a chemokine by recruiting neutrophils to sites of infection and activating them to eliminate pathogens by phagocytosis and by production of oxygen radicals and nitric oxide. Previous studies have shown that LT may achieve lower IL-8 expression in two ways: through mRNA destabilization [17] and by preventing histone H3 phosphorylation at Ser 10 thus altering chromatin accessibility of IL-8 promoter to NF-κB in epithelial cells [35].

Both the anti-PA and anti-LF mAbs tested by us in the TN assay were able to neutralise LT effectively. Human mAbs, IQNLF and IQNPA, were shown to be potent inhibitors of LT activity. IQNPA specifically recognises domain IV of PA, thus impeding the binding of PA to its cell surface receptors. IQNLF binds to domain I of LF and may prevent interaction of LF with PA [9]. We examined whether these antibodies could act together when combined to inhibit LT activity in endothelial cells. The potency of these mAbs increased significantly when they were combined in the TN assay indicating a possible synergistic effect. Previously an additive effect was reported in the mouse macrophage assay [9], suggesting that LT and the mAbs interact differently with human endothelial cells than with mouse macrophages. LT activates the inflammasome of macrophages from several mouse and rat strains, through the NOD-like receptor protein (Nlrp) 1b, leading to rapid cell death as a result of capase-1 production [36,37]. This activity was found to be absent in human macrophages, possibly due to a different *Nlrp1b* type in humans [37]. As LT acts differently on mouse and human cell lines the TN assays based on mouse macrophages and human cells are likely to give different outcomes following exposure of cells to LT or to a mixture of LT and TN mAbs. 

The mouse mAb 9A11 was also demonstrated to be a potent LT-neutralising Ab. 9A11 differs in its mode of action from IQNLF and binds to two different epitopes within the LF zinc-binding domain involved in LF substrate recognition, effectively blocking enzymatic activity [38].

Taken together these changes induced by LT in HUVEC jr2 cells may contribute to dysfunctional endothelial cell stress which will lead to barrier dysfunction and vascular leakage and ultimately will compromise the integrity of blood vessels. 

We conclude that the use of human endothelial cells in this *in vitro* TN assay model is a valid alternative to mouse macrophage assay and that it provides a more biologically relevant representation of events that characterise the interaction between LT and the human host. We accept that comparisons between our assay and the mouse macrophage TN assay may not be straight forward. However we believe that this assay will enable us to determine the counter effects of TN monoclonal antibody preparations on a different level, which will aid our understanding of the neutralising capabilities of therapeutic mAbs.

## References

[1] Collier R.J., Young J.A. (2003). Anthrax toxin. Ann. Rev. Cell Dev. Biol..

[2] Moayeri M., Leppla S.H. (2004). The roles of anthrax toxin in pathogenesis. Curr. Opin. Microbiol..

[3] Leppla S. (1982). Anthrax toxin edema factor: a bacterial adenylate cyclase that increases cyclic AMP concentrations of eukaryotic cells. Proc. Natl. Acad. Sci. USA.

[4] Duesbery N.S., Webb C.P., Leppla S.H., Gordon V.M., Klimpel K.R., Copeland T.D., Ahn N.G., Oskarsson M.K., Fukasawa K., Paul K.D. (1998). Proteolytic inactivation of MAP-kinase-kinase by anthrax lethal factor. Science.

[5] Bardwell A.J., Abdollahi M., Bardwell L. (2004). Anthrax lethal factor-cleavage products of MAPK (mitogen activated protein kinase) kinases exhibit reduced binding to their cognate MAPKs. Biochem. J..

[6] Chopra A.P., Boone S.A., Liang X., Duesbery N.S. (2003). Anthrax lethal factor proteolysis and inactivation of MAPK kinase. J. Biol. Chem..

[7] Matheny J., Mair M., Smith B. (2008). Cost/success projections for US biodefense countermeasure development. Nat. Biotechnol..

[8] Chen Z., Moayeri M., Purcell R. (2011). Monoclonal antibody therapies against anthrax. Toxins.

[9] Albrecht M.T., Li H., Williamson E.D., LeButt C.S., Flick-Smith H.C., Quinn C.P., Westra H., Galloway D., Mateczun A., Goldman S. (2007). Human monoclonal antibodies against anthrax lethal factor and protective antigen act independently to protect against *Bacillus anthracis* infection and enhance endogenous immunity to anthrax. Infect. Immun..

[10] Staats H.F., Alam S.M., Scearce R.M., Kirwan S.M., Zhang J.X., Gwinn W.M., Haynes B.F. (2007). *In vitro* and *in vivo* characterization of anthrax anti-protective antigen and anti-lethal factor monoclonal antibodies after passive transfer in a mouse lethal toxin challenge model to define correlates of immunity. Infect. Immun..

[11] Friedlander A.M., Bhatnagar R., Leppla S.H., Johnson L., Singh Y. (1993). Characterization of macrophage sensitivity and resistance to anthrax lethal toxin. Infect. Immun..

[12] Hering D., Thompson W., Hewetson J., Little S., Norris S., Pace-Templeton J. (2004). Validation of the anthrax lethal toxin neutralization assay. Biologicals.

[13] Pittman P.R., Kim-Ahn G., Pifat D.Y., Coonan K., Gibbs P., Little S., Pace-Templeton J.G., Myers R., Parker G.W., Friedlander A.M. (2002). Anthrax vaccine: immunogenicity and safety of a dose-reduction, route-change comparison study in humans. Vaccine.

[14] Zmuda J.F., Zhang L., Richards T., Pham Q., Zukauskas D., Pierre J.L., Laird M.W., Askins J., Choi G.H. (2005). Development of an edema factor-mediated cAMP-induction bioassay for detecting antibody-mediated neutralization of anthrax protective antigen. J. Immunol. Methods.

[15] Barson H.V., Mollenkopf H., Kaufmann S.H.E., Rijpkema S. (2008). *In vitro* exposure to anthrax lethal toxin suppresses chemokine production in the human neutrophil-like cell line NB-4. Biochem. Biophy. Res. Commun..

[16] Wheeler J.X., Whiting G., Rijpkema S. (2007). Proteomic analysis of the response of the human neutrophil-like cell line NB-4 after exposure to anthrax lethal toxin. Proteomics Clin. Appl..

[17] Batty S., Chow E.M.C., Kassam A., Der S.D., Mogridge J. (2006). Inhibition of mitogen-activated protein kinase signaling by *Bacillus anthracis* lethal toxin causes destabilization of interleukin-8 mRNA. Cell Microbiol..

[18] During R.L., Li W., Hao B., Koenig J.M., Stephens D.S., Quinn C.P., Southwick F.S. (2005). Anthrax lethal toxin paralyzes neutrophil actin-based motility. J. Infect. Dis..

[19] During R.L., Gibson B.G., Li W., Bishai E.A., Sidhu G.S., Landry J., Southwick F.S. (2007). Anthrax lethal toxin paralyzes actin-based motility by blocking Hsp27 phosphorylation. EMBO J..

[20] Fang H., Cordoba-Rodriguez R., Lankford C.S., Frucht D.M.J. (2005). Anthrax lethal toxin blocks MAPK kinase-dependent IL-2 production in CD4 + T cells. J. Immunol..

[21] Popov S.G., Villasmil R., Bernardi J., Grene E., Cardwell J., Popova T., Wu A., Alibek D., Bailey C., Alibek K. (2002). Effect of *Bacillus anthracis* lethal toxin on human peripheral blood mononuclear cells. FEBS Lett..

[22] Rolando M., Stefani C., Flatau G., Auberger P., Mettouchi A., Mhlanga M., Rapp U., Galmiche A., Lemichez E. (2010). Transcriptome dysregulation by anthrax lethal toxin plays a key role in induction of human endothelial cell cytotoxicity. Cell Microbiol..

[23] Stebbings R., Findlay L., Edwards C., Eastwood D., Bird C., North D., Mistry Y., Dilger P., Liefooghe E., Cludts I. (2007). Cytokine storm in the phase I trial of monoclonal antibody TGN1412: Better understanding the causes to improve preclinical testing of immunotherapeutics. J. Immunol..

[24] Fleck R.A., Athwal H., Bygraves J.A., Hockley D.J., Feavers I.M., Stacey G.N. (2003). Optimization of NB-4 and HL-60 differentiation for use in opsonophagocytosis assays. In Vitro Cell Dev. Biol. Anim..

[25] Combistats computer program for the statistical analysis of data from biological dilution assays or potency assays (EDQM).. http://combistats.edqm.eu/.

[26] Erwin J.J., DaSilva L.M., Bavari S., Little S.F., Friedlander A.M., Chanh T.C. (2001). Macrophage-derived cell lines do not express proinflammatory cytokines after exposure to *Bacillus anthracis* lethal toxin. Infect. Immun..

[27] Warfel J.M., D’Agnillo F. (2008). Anthrax lethal toxin enhances TNF-Induced endothelial VCAM-1 expression via an IFN regulatory factor-1-dependent mechanism. J. Immunol..

[28] Guarner J., Jernigan J.A., Shieh W.J., Tatti K., Flannagan L.M., Stephens D.S., Popovic T., Ashford D.A., Perkins B.A., Zaki S.R. (2003). Pathology and pathogenesis of bioterrorism-related inhalational anthrax. Am. J. Pathol..

[29] Moayeri M., Haines D., Young H.A., Leppla S.H. (2003). *Bacillus anthracis* lethal toxin induces TNF-alpha-independent hypoxia-mediated toxicity in mice. J. Clin. Invest..

[30] Park J.M., Ng V.H., Maeda S., Rest R.F., Karin M. (2004). Anthrolysin O and other Gram-positive cytolysins are Toll-like receptor 4 agonists. J. Exp. Med..

[31] van Sorge N.M., Ebrahimi C.M., McGillivray S.M., Quach D., Sabet M., Guiney D.G., Doran K.S. (2008). Anthrax toxins inhibit neutrophil signaling pathways in brain endothelium and contribute to the pathogenesis of meningitis. PLoS One.

[32] Warfel J.M., Steele A.D., D’Agnillo F. (2005). Anthrax lethal toxin induces endothelial barrier dysfunction. Am. J. Pathol..

[33] Posern G., Treisman R. (2006). Actin’ together: Serum response factor, its cofactors and the link to signal transduction. Trends Cell. Biol..

[34] Steele A.D., Warfel J.M., D’Agnillo F. (2005). Anthrax lethal toxin enhances cytokine-induced VCAM-1 expression on human endothelial cells. Biochem. Biophys. Res. Commun..

[35] Raymond B., Batsche E., Boutillon F., Wu Y-Z., Leduc D., Balloy V., Raoust E., Muchardt C., Goossens P.L., Touqui L. (2009). Anthrax lethal toxin impairs IL-8 expression in epithelial cells through Inhibition of histone H3 modification. PLoS Pathog..

[36] Moayeri M., Crown D., Newman Z.L., Okugawa S., Eckhaus M., Cataisson C., Liu S., Sastalla I., Leppla S.H. (2010). Inflammasome sensor Nlrp1b-dependent resistance to anthrax is mediated by Caspase-1, IL-1 signaling and neutrophil recruitment. PLoS Pathog..

[37] Newman Z.L., Crown D., Leppla S.H., Moayeri M. (2010). Anthrax lethal toxin activates the inflammasome in sensitive rat macrophages. Biochem. Biophys. Res. Commun..

[38] Nguyen M.L., Terzyan S., Ballard J.D., James J.A., Farris A.D. (2009). The major neutralizing antibody responses to recombinant anthrax lethal and edema factors are directed to non-cross-reactive epitopes. Infect. Immun..

